# Group 2 Innate Lymphoid Cells Are Detrimental to the Control of Infection with *Francisella tularensis*

**DOI:** 10.4049/jimmunol.2100651

**Published:** 2023-01-16

**Authors:** Joshua Dow, Urszula M. Cytlak, Joshua Casulli, Craig P. McEntee, Catherine Smedley, Suzanne H. Hodge, Riccardo V. D’Elia, Matthew R. Hepworth, Mark A. Travis

**Affiliations:** * Lydia Becker Institute for Immunology and Inflammation, Manchester, United Kingdom;; † Wellcome Trust Centre for Cell-Matrix Research, Manchester, United Kingdom;; ‡ Faculty of Biology, Medicine and Health, Manchester Academic Health Sciences Centre, University of Manchester, Manchester, United Kingdom;; § Targeted Therapy Group, Division of Cancer Sciences, Manchester, United Kingdom;; ¶ Defence Science and Technology Laboratory, Porton Down, Salisbury, United Kingdom; and; ‖ Strathclyde Institute of Pharmacy & Biomedical Sciences, University of Strathclyde, Glasgow, United Kingdom

## Abstract

Innate lymphoid cells (ILCs) are capable of rapid response to a wide variety of immune challenges, including various respiratory pathogens. Despite this, their role in the immune response against the lethal intracellular bacterium *Francisella tularensis* is not yet known. In this study, we demonstrate that infection of the airways with *F. tularensis* results in a significant reduction in lung type 2 ILCs (ILC2s) in mice. Conversely, the expansion of ILC2s via treatment with the cytokine IL-33, or by adoptive transfer of ILC2s, resulted in significantly enhanced bacterial burdens in the lung, liver, and spleen, suggesting that ILC2s may favor severe infection. Indeed, specific reduction of ILC2s in a transgenic mouse model results in a reduction in lung bacterial burden. Using an in vitro culture system, we show that IFN-γ from the live vaccine strain–infected lung reduces ILC2 numbers, suggesting that this cytokine in the lung environment is mechanistically important in reducing ILC2 numbers during infection. Finally, we show Ab-mediated blockade of IL-5, of which ILC2s are a major innate source, reduces bacterial burden postinfection, suggesting that IL-5 production by ILC2s may play a role in limiting protective immunity. Thus, overall, we highlight a negative role for ILC2s in the control of infection with *F. tularensis*. Our work therefore highlights the role of ILC2s in determining the severity of potentially fatal airway infections and raises the possibility of interventions targeting innate immunity during infection with *F. tularensis* to benefit the host.

## Key Points

- Enhancing ILC2 numbers increases *Francisella tularensis* bacterial burden.
- Reducing ILC2 numbers promotes *Francisella tularensis* bacterial clearance.
- IFN-γ reduces ILC2s, with ILC2 production of IL-5 detrimental to bacterial clearance.

## Introduction

F*rancisella tularensis* is a highly infectious intracellular bacterium and the causative agent of tularemia (1). Capable of extensive replication and dissemination to peripheral organs (2), respiratory infection with *F. tularensis* can cause lethal disease in the absence of intervention (3). Moreover, the enhanced virulence of the bacterium via this route has led to concerns that *F. tularensis* could be used as a biological weapon if aerosolized (4, 5).

Much of our current knowledge of the early immune response against *F. tularensis* has focused on the role of myeloid cells (6). For example, macrophages and neutrophils serve as sites of extensive replication during respiratory infection, with dysregulation of these mechanisms proving detrimental to the host immune response (7–9). In contrast, the contributions of innate lymphocyte responses in the pathogenesis or control of infection with *F. tularensis* are less understood. Innate lymphoid cells (ILCs) are tissue-resident innate lymphocytes, which are capable of a rapid response to a wide variety of immunological challenges. ILCs are divided into three major subsets that is, ILC1s, ILC2s, and ILC3s, which are largely analogous to CD4^+^ Th cell subsets in their phenotype and effector functions (10). ILCs are frequently found at barrier sites, such as the skin, intestine, and the lung and play critical roles in early protection against invading pathogens (10). At steady state, ILC2s represent the dominant ILC subset in the murine lung (11), playing key roles in the immune response against several respiratory pathogens (12–14). However, the role of ILCs during respiratory infection with *F. tularensis* is currently unknown. Thus, to address this gap, we used a murine model of infection with a live vaccine strain (LVS) of *F. tularensis*. This strain has been instrumental in the study of tularemia, as it represents a less virulent strain in humans when compared with other strains such as Schu S4 (1), while retaining its lethality in mice when administered via the intranasal (i.n.) route (15).

In this study, we demonstrate that *F. tularensis* LVS infection is associated with a loss of ILC2s from the lungs. Moreover, we reveal that this reduction in ILC2s may benefit the host immune response against the bacteria, as, conversely, expansion of ILC2 numbers by either IL-33 treatment or adoptive transfer of ILC2s causes significantly exacerbated bacterial burdens in the lung, liver, and spleen of mice. In further support of a reduction in ILC2s being beneficial for host immunity, depletion of ILC2s using a transgenic mouse model resulted in reduced bacterial burden in the lung. Mechanistically, we show that IFN-γ from the LVS-infected lung appears to drive the infection-induced reduction in ILC2s. Finally, blockade of IL-5, of which ILC2s are a major innate source, reduces bacterial burden, suggesting that ILC2-produced IL-5 may limit protective host immunity. Overall, our data highlight a negative role for ILC2s in the control of infection with *F. tularensis* LVS. We therefore postulate that manipulation of ILC2 numbers and cytokines during early infection with *F. tularensis* could be of potential therapeutic benefit.

## Materials and Methods

### Bacterial strains

*F. tularensis* LVS was derived from an original NDBR101 *Pasteurella tularensis* live vaccine, experimental lot 4. The vaccine contained 6 × 10^9^ CFU of lyophilized *F. tularensis* LVS, which was stored in a culture collection at −20°C at the Defense Science and Technology Laboratory. Master stocks and i.n. challenge doses were prepared as previously described (9).

### Mice and in vivo *F. tularensis* infection

Female C57BL/6 mice (Charles River, Margate, U.K.), Rag1^−/−^ mice on a C57BL/6 background (16), inducible ICOS–diphtheria toxin receptor (DTR) (iCOS-T) mice on a C57BL/6 background (17), originally a gift from Dr. Andrew McKenzie (MRC Laboratory of Molecular Biology, Cambridge, U.K.), and Red-5 mice (Red5Cre (B6(C)-*Il5^tm1.1(icre)Lky^*/J, stock number 030926), purchased from The Jackson Laboratory and originally generated by Prof. Richard Locksley, University of California San Francisco [18]) were kept in specific pathogen-free conditions according to institutional and U.K. Home Office guidelines in the Biological Services Unit at The University of Manchester. Eight- to 12-wk-old mice were infected i.n. with 50 μl of PBS containing ∼1000 CFU of *F. tularensis* LVS. An infectious dose was confirmed by CFU count on blood cysteine glucose agar plates. All procedures were performed in accordance with the Home Office Scientific Procedures Act (1986) and under the Department for Environment, Food and Rural Affairs license. Survival of mice was defined as mice reaching the defined humane endpoint as specified by our license granted by the U.K. Home Office. For all experiments, the humane endpoint was defined as either weight loss in excess of 25% of starting body weight, and/or loss of mobility and condition, as determined by use of a clinical scoring system that monitored the general condition of mice throughout experiments (9).

### Depletion of ILC2s in iCOS-T mice

iCOS-T mice, which allow specific deletion of ILC2s via injection of DT (Sigma-Aldrich) (17), were treated via i.p. injection daily with 1 µg of DT/mouse. Mice were treated daily from 4 d prior to i.n. infection with *F. tularensis* LVS, and daily postinfection (p.i.) until mice were culled for analysis.

### Treatment of mice with anti–IL-5 Ab

C57BL/6 mice were treated i.p. with 100 µg/mouse anti–IL-5 Ab (clone TRFK5) or rat IgG1 isotype control (anti-HRP) (both from Bio X Cell). Mice were treated every other day from 4 d prior to infection prior to i.n. infection with *F. tularensis* LVS, and every other day p.i. until mice were culled for analysis.

### Enumeration of bacterial burden

Bacterial burdens were enumerated from the lung, liver, and spleen of mice, with samples processed <2 h postmortem. All organs were collected in PBS and weighed. Samples were disrupted through a 40-μm cell sieve, followed by serial dilution of organ homogenates in PBS. Although the use of PBS in this step will not cause extensive cell lysis, this method was used to ensure that cells were available for downstream flow cytometry analysis and gave a consistently robust measure of bacterial CFU in organs. Bacteria were incubated for 4–5 d at 37°C, and once grown sufficiently, single colonies were counted and organ weights were used to express data as CFU per gram of organ.

### Digestion of lung tissue

Lung tissue was cut into small sections and shaken in an incubator at 37°C in PBS containing 3 mg/ml collagenase D (Roche) for 40 min. After digest, tissues were disrupted through a 40-μm cell sieve and samples washed with PBS. RBCs were lysed using RBC lysing buffer Hybri-Max (Merck), washed again in PBS, and cell numbers were counted before staining and analysis by flow cytometry.

### Flow cytometry

Samples were stained with fluorophore-labeled Abs against the following: CD3 (clone 145-2C11), CD5 (clone 53-7.3), CD45 (clone 30-F11), CD64 (clone X54-5/7.1), CD90.2 (clone 30-H12), CD127 (clone A7R34), Ly6G (clone 1A8), NK1.1 (clone PK136), T-bet (clone 4B10) (BioLegend), B220 (clone RA3-6B2), CD4 (clone RM4.5), CD11b (clone M1/70), CD11c (clone N418), Foxp3 (clone FJK-16s), KLRG1 (clone 2F1), IL-13 (clone eBio13A), RORγt (clone B2D), Siglec-F (clone 1RNM44N), ST2 (clone RMST2-33), streptavidin (no. 17-4317-82) (eBioscience), and streptavidin (no. 564176) (BD Biosciences). Dead cells were excluded from analysis using an Aqua Live/Dead viability stain (Invitrogen). Abs were diluted to desired concentrations in FACS buffer (2% FCS, 2 mM EDTA in 1× PBS) for targeting of cell surface markers, and 1× permeabilization buffer (concentrate/dH_2_O, 1:9) (eBioscience) for intracellular markers. First, cells were stained with Abs against cell surface markers for 30 min at 4°C. Where appropriate, cells were washed, centrifuged, and stained with a streptavidin Ab for 15 min at 4°C. For intracellular staining, samples were then washed and resuspended in fixation/permeabilization buffer (concentrate/diluent, 1:3) (eBioscience), incubated overnight at 4°C, washed in 1× permeabilization buffer, and then incubated for 45 min at 4°C with Abs against intracellular markers.

For cytokine staining, lung cells were treated with stimulating mixture containing PMA, ionomycin, and protein transport inhibitors (eBioscience, 00-4975-03), at 1:500 in RPMI 1640 containing 10% FCS, for 4 h at 37°C, with moderate agitation (shaking incubator, 400 rpm) before staining as above.

Gating was established using either fluorescence minus one or isotype controls. Prior to data acquisition, cells were washed in FACS buffer and stored at 4°C until analysis on a BD LSRFortessa (BD Biosciences). Data were analyzed using FlowJo software version 10.7.1 (Tree Star, Ashland, OR).

### Administration of IL-33

Recombinant murine IL-33 (rIL-33; carrier-free, R&D Systems) was administered to mice via the i.p. route at a dose of 0.5 μg/mouse every 2 d starting 5 d before infection with *F. tularensis* LVS. Control mice were administered vehicle (PBS).

### I.n. transfer of ILC2s

Naive C57BL/6 mice were given four doses of rIL-33 as above (i.p.; 0.5 μg/mouse), before being culled 2 d after the final dose. Lung ILC2s (live CD45^+^Lin^−^CD90.2^mid/hi^CD127^+^KLRG1^+^) were then sort-purified, and 1 × 10^5^ ILC2s were transferred via the i.n. route into LVS-infected mice at day 3 p.i. Control non-transfer mice were given PBS. All mice in these studies were subsequently culled at day 7 p.i.

### In vitro ILC2 culture

Lung ILC2s were sort-purified as above, and cells were transferred to 96-well plates in 200 μl of complete RPMI 1640 at 1 × 10^5^ ILC2s per well. Cells were then cultured for 7 d with IL-7 (Invitrogen) at 25 ng/ml. Naive and LVS-infected lung supernatants were generated by 24-h incubation of homogenized lung samples at 37°C in complete RPMI 1640. Cell-free supernatants were collected by centrifugation and filtration through a sterile syringe 0.22-μm microfilter. Then, 100 μl of supernatants was then added to 1 × 10^5^ ILC2s (in 100 µl) and cultured for 5 d. All wells were cultured with IL-7 at 25 ng/ml and, where indicated, anti–IFN-γ Ab (clone XMG1.2, Bio X Cell) was added to culture media at 10 μg/ml. For live ILC2 cell counts, a hemocytometer counting chamber was used, with live ILC2 cell counts enumerated via trypan blue dye exclusion.

### Statistical analysis

All graphs and statistical analyses were produced using GraphPad Prism 9. Normality of data was determined using the Shapiro–Wilk normality test, with appropriate parametric and nonparametric statistical tests performed for each dataset (stated in figure legends). Data were expressed as mean ± SEM. Statistical significance was considered at *p* < 0.05.

## Results

### ILC2s are reduced during infection with *F. tularensis* LVS

**FIGURE 1.**
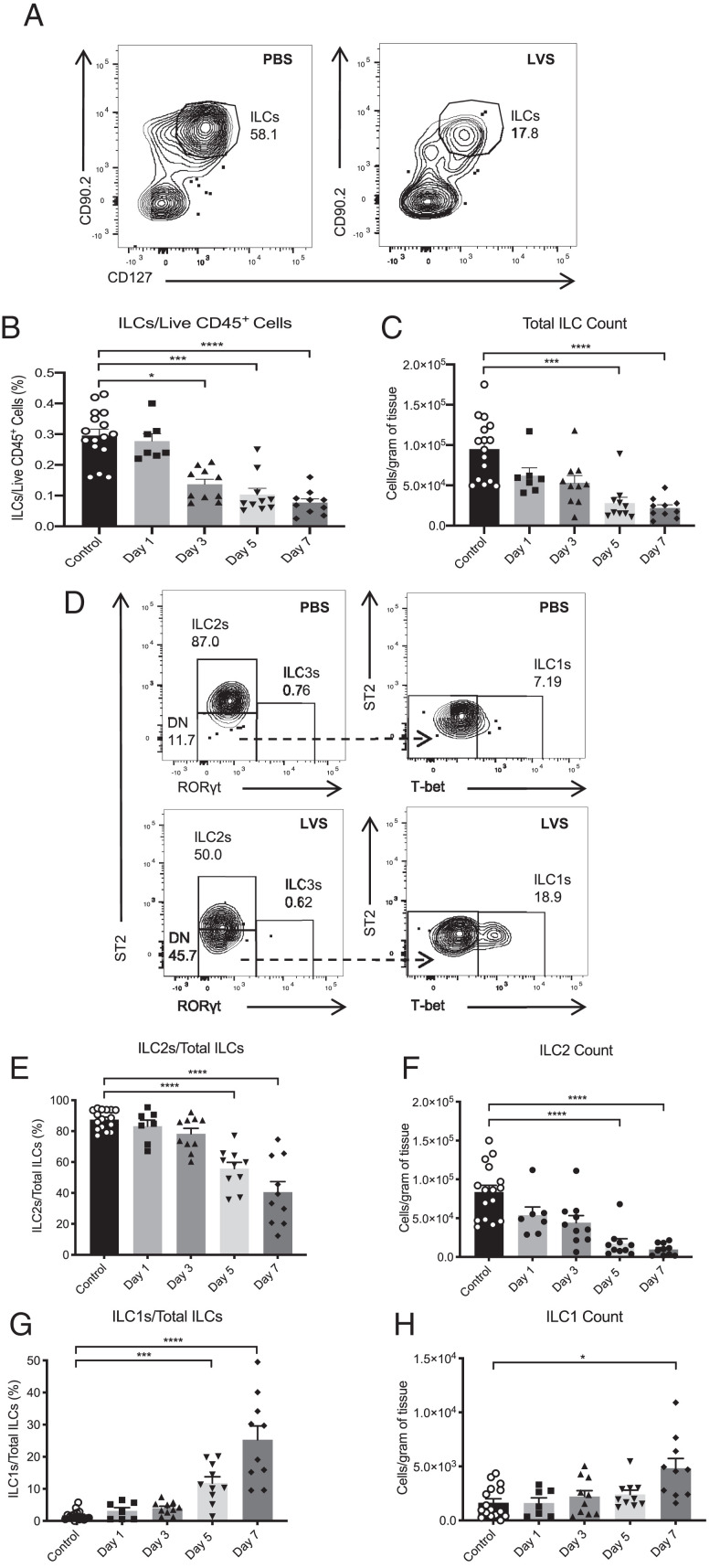
ILC2s are decreased in the lung of C57BL/6 mice during infection with *F. tularensis* LVS. (**A**) Representative plots for identification of ILCs as CD127^+^CD90.2^+^ cells in PBS control and LVS-infected C57BL/6 (WT) mice (plot from day 5 p.i.). Cells displayed in the plots were previously identified as live CD45^+^Lin^−^ (i.e., CD3, CD5, CD11b, CD11c, B220, NK1.1). (**B** and **C**) Total ILC numbers were quantified and expressed as (B) a percentage of live CD45^+^ cells and (C) cell counts per gram of tissue. (**D**) ILC subsets were gated from CD127^+^CD90.2^+^ cells (depicted in A). ILC2s (left plots) were identified as ST2^+^RORγt^−^ cells. ILC1s were gated from the ST2^−^RORγt^−^ population (DN in left plots) and subsequently identified as T-bet^+^ cells (right plots). (**E**–**H**) These subsets were then quantified and expressed as a frequency of total ILCs as well as cell counts per gram of tissue (E and F for ILC2s, G and H for ILC1s). Data represent six or two to three independent experiments for control and infected mice, respectively (*n* = 16 for control, *n* = 7 for day 1, *n* = 10 for days 3, 5, and 7 p.i.). Statistical analysis was performed using (B, C, and F–H) a Kruskal–Wallis test for nonparametric data, and (E) one-way ANOVA for parametric data, with Dunn’s and Holm–Sidak’s multiple comparison tests performed for each method of analysis, respectively. *p < 0.05, ***p < 0.001, ****p < 0.0001.

To define the ILC response during pulmonary infection with *F. tularensis* LVS, ILCs were identified in the lung and defined by a lack of expression of lineage markers (Lin^−^) and high expression of CD90.2 and CD127 (Fig. 1A; full gating strategy is shown in Supplemental Fig. 1). Interestingly, we observed a significant and continual decrease in total lung ILCs during the course of infection, both in frequency and absolute cell numbers (Fig. 1B, 1C).

To determine whether this reduction in total ILC numbers was a result in changes to a specific ILC subset, we further analyzed specific subgroups of ILCs. As previously reported (12), we found that ILC2s (defined as ST2^+^) represented the dominant lung ILC subset under steady-state conditions, with a small number of ILC1s (T-bet^+^) present (Fig. 1D). In contrast, ILC3s (RORγt^+^) were virtually absent from the airways in both naive and infected mice (Fig. 1D). Upon infection with *F. tularensis* LVS, both the frequency and total number of ILC2s were significantly reduced from day 5 p.i. (Fig. 1D–F). This subset-specific reduction was also associated with a smaller but reproducible concurrent increase in ILC1 numbers (Fig. 1D, 1G, 1H). Overall, these data indicate that pulmonary infection with *F. tularensis* LVS results in significant perturbation and reduction in the lung ILC compartment during the later stages of infection, predominantly due to a reduction in ILC2s.

### IL-33–induced expansion of the lung ILC2 compartment significantly enhances bacterial burden

**FIGURE 2.**
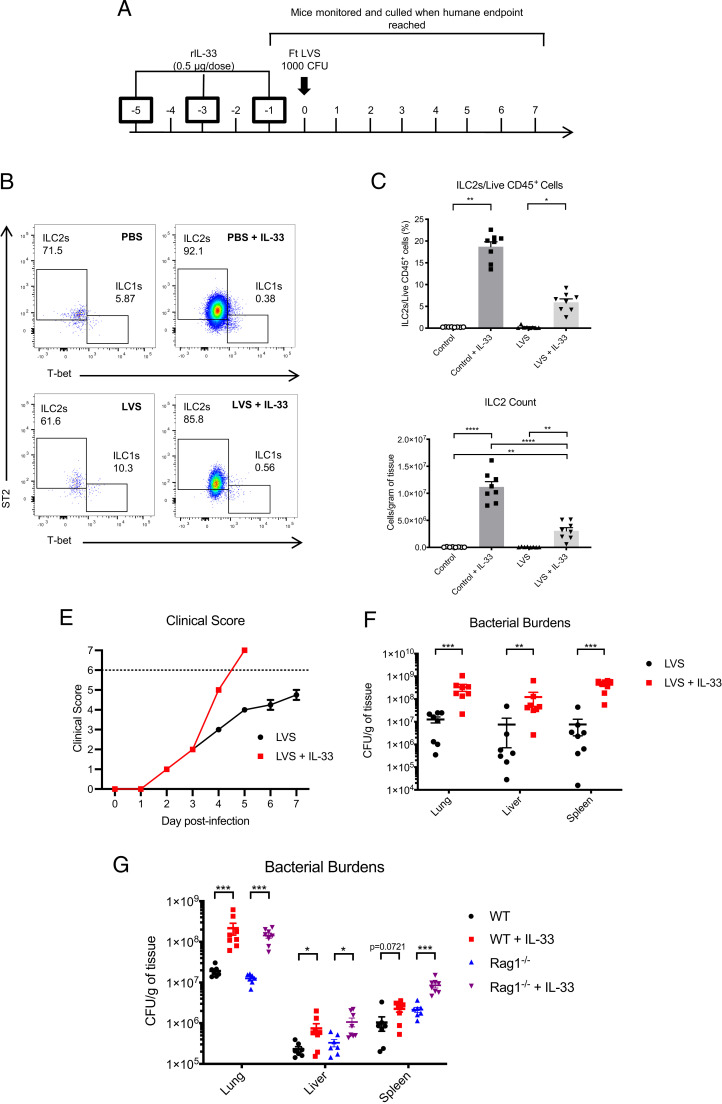
IL-33 treatment is detrimental to the control of infection with *F. tularensis* LVS in the absence of lymphocytes. (**A**) C57BL/6 WT mice were treated as indicated. (**B**) Representative plots for identification of ILC2s (live CD45^+^Lin^−^CD127^+^ cells) in untreated or IL-33–treated mice during infection with *F. tularensis* LVS. (**C** and **D**) ILC2s (ST2^+^) were quantified and expressed as a frequency of live CD45^+^ cells as well as cell counts per gram of tissue. (**E**) Clinical scores of LVS-infected mice with or without IL-33 treatment, with dotted line indicating the humane endpoint of the experiment (see *Materials and Methods*). (**F**) Bacterial burdens were enumerated in the lung, liver, and spleen of mice at day 4 p.i. (**G**) WT and Rag1^−/−^ mice were treated as indicated in (A), and bacterial burdens were enumerated in the lung, liver, and spleen of mice at day 4 p.i. Data represent two independent experiments (*n* ≥ 7). Statistical analysis was performed using (C) a Kruskal–Wallis test with Dunn’s correction for multiple comparisons, (D) one-way ANOVA with Holm–Sidak’s correction for multiple comparisons, (F) an unpaired *t* test, or (G) a Mann–Whitney *U* test. *p < 0.05, **p < 0.01, ***p < 0.001, ****p < 0.0001.

ILC2s have previously been shown to be involved in the immune response against other intracellular infections (12, 13) and tissue repair p.i. (12). Thus, it was hypothesized that the subset-specific reduction in ILC2s could be advantageous to the growth and pathogenesis of *F. tularensis* LVS. To address how changes in ILC2s impact the progress of *F. tularensis* infection, mice were treated with IL-33 prior to i.n. infection (see (Fig. 2A for treatment regimen). As expected (10), IL-33 treatment caused a significant expansion of ILC2s, both by percentage and total numbers, in the naive mouse lung (Fig. 2B–D). This expansion was also observed in the infected lung, albeit to a lesser extent than in IL-33–treated uninfected mice (Fig. 2B–D), suggesting that infection antagonized the ILC2 response even following IL-33–induced expansion.

We next tested how IL-33 treatment affected pathogen burdens and infectious outcome. Surprisingly, IL-33 treatment resulted in enhanced morbidity in mice infected with *F. tularensis* LVS, which required euthanasia of animals by day 4 p.i. under animal license restrictions (Fig. 2E). Moreover, treatment with IL-33 resulted in significantly enhanced bacterial burdens in the lung, liver, and spleen at day 4 p.i. (Fig. 2F). Taken together, these data indicate that IL-33 treatment is detrimental to the outcome of infection with *F. tularensis* LVS.

Our findings showed that the IL-33 treatment could exacerbate infection in the host, potentially via ILC2 expansion. However, as IL-33 is a pleiotropic cytokine that can affect other immune cells (18, 19), it was important to consider the wider impact of IL-33 treatment on the immune system during *F. tularensis* infection. In line with ILC2 expansion and prior reports (18), we found that IL-33 treatment caused enhancement of eosinophil numbers (Supplemental Fig. 2A–C). Furthermore, type 2 CD4^+^ST2^+^ T cell (Supplemental Fig. 2D, 2E) and CD4^+^Foxp3^+^ regulatory T cell numbers were also enhanced during infection (Supplemental Fig. 2F, 2G). Thus, to address whether the effects of IL-33 treatment were acting via enhancement of innate or adaptive immune responses, we used Rag1^−/−^ mice that lack adaptive immune cells. We observed that, even in the absence of adaptive immunity, IL-33 induced a significant increase in bacterial burdens in Rag1^−/−^ mice in the lung, liver, and spleen to the same extent to that seen in wild-type (WT) mice (Fig. 2G), with both ILC2s and eosinophils still significantly expanded in IL-33–treated LVS-infected Rag1^−/−^ mice (Supplemental Fig. 3A, 3B). Thus, these data strongly indicated that the innate immune response was sufficient to promote IL-33–mediated enhancement of bacterial burdens during *F. tularensis* LVS infection.

**FIGURE 3.**
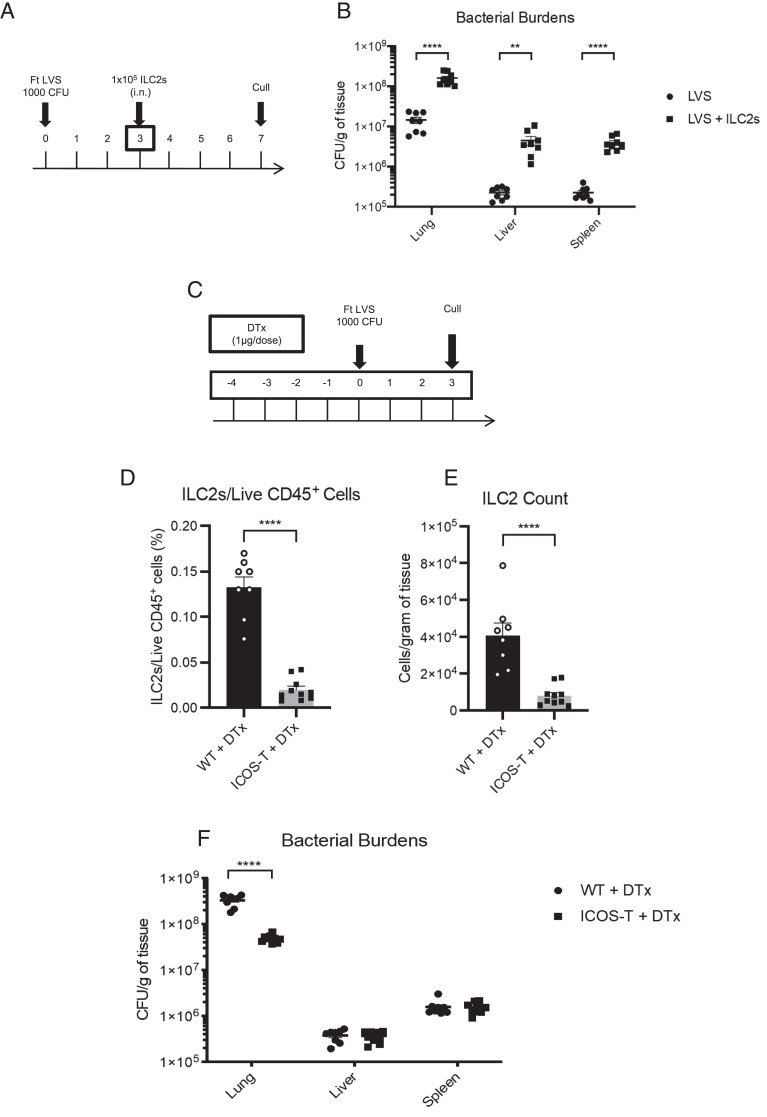
Enhanced bacterial burdens p.i. with *F. tularensis* LVS are induced by direct transfer of ILC2s to the lung, and reduction of ILC2 numbers reduces bacterial burden. (**A**) C57BL/6 WT mice were infected with 1000 CFU of *F. tularensis* LVS and received 1 × 10^5^ FACS-sorted live CD45^+^Lin^−^CD90.2^mid/hi^CD127^+^KLRG1^+^ i.n. at day 3 p.i. Control (LVS) mice were given an i.n. dose of 50 μl of PBS. (**B**) Bacterial burdens (CFU per gram of organ) were enumerated at day 7 p.i. (**C**–**F**) iCOS-T mice (17) or littermate controls without Cre or DTR expression (WT) were injected with DT and infected with 1000 CFU of *F. tularensis* LVS. (C) Schematic of DT treatment and *F. tularensis* LVS infection timings. (D and E) Frequency as a percentage of live CD45^+^ cells (D) and absolute cell number of (E) ILC2s in the lung of infected control and iCOS-T mice at day 3 p.i. (F) Bacterial burden in lung, liver, and spleen of control and iCOS-T mice at day 3 p.i. Data represent two independent experiments (*n* = 7–10 mice). Statistical analysis was performed using a Mann–Whitney *U* test (spleen in F) and unpaired *t* test (B, D, E, and lung and liver in F). **p < 0.01, ****p < 0.0001.

Next, to directly test the role for enhanced lung ILC2s to the outcome of infection, sort-purified ILC2s were transferred to WT mice via the i.n. route at day 3 p.i. (Fig. 3A). Strikingly, transfer of ILC2s also resulted in significantly enhanced bacterial burdens in the lung, liver, and spleen (Fig. 3B), similar in magnitude to that observed when mice were treated with IL-33 (Fig. 2F). Thus, together, these data strongly suggest that ILC2s are detrimental to the control of infection with *F. tularensis* LVS.

### Depletion of ILC2 numbers significantly reduces pulmonary bacterial burdens after *F. tularensis* LVS infection

The data above show that promoting ILC2 numbers, either via injection of IL-33 or direct transfer of ILC2s to the lung, causes enhanced bacterial burden. These data therefore suggest the possibility that depletion of ILC2s may favor reduced bacterial burdens p.i. To directly test this possibility, we used iCOS-T mice, which allow selective depletion of ILC2s after injection of DT while retaining iCOS^+^CD4^+^ T cells due to *Cd4*^Cre^-mediated excision of the DTR locus (17). iCOS-T mice or littermate controls without Cre or DTR expression (referred to as WT) were injected i.p with DT daily from 4 d prior to infection and during infection (Fig. 3C). This led to a reduction in the percentage and absolute numbers of ILC2s in the lung (Fig. 3D, 3E), with no reductions in CD4^+^ T cell or ILC1 percentage or absolute numbers (Supplemental Fig. 3C–F). Reduction of ILC2s following DT treatment led to a significant reduction in bacterial burden seen in the lung of iCOS-T mice, although we did not observe altered systemic bacterial burdens in spleen and liver using this inducible model (Fig. 3F). Thus, these data suggest that reducing ILC2 numbers may be a strategy for reducing bacterial burden locally in the lung after respiratory *F. tularensis* infection.

### IFN-γ in the infected lung environment is important in driving infection-induced reduction in ILC2 numbers

To dissect the mechanistic basis of the observed decrease in ILC2s during *F. tularensis* LVS infection we next considered whether a soluble mediator present in the *F. tularensis* LVS-infected lung environment could be important in driving the reduction in ILC2 numbers.

**FIGURE 4.**
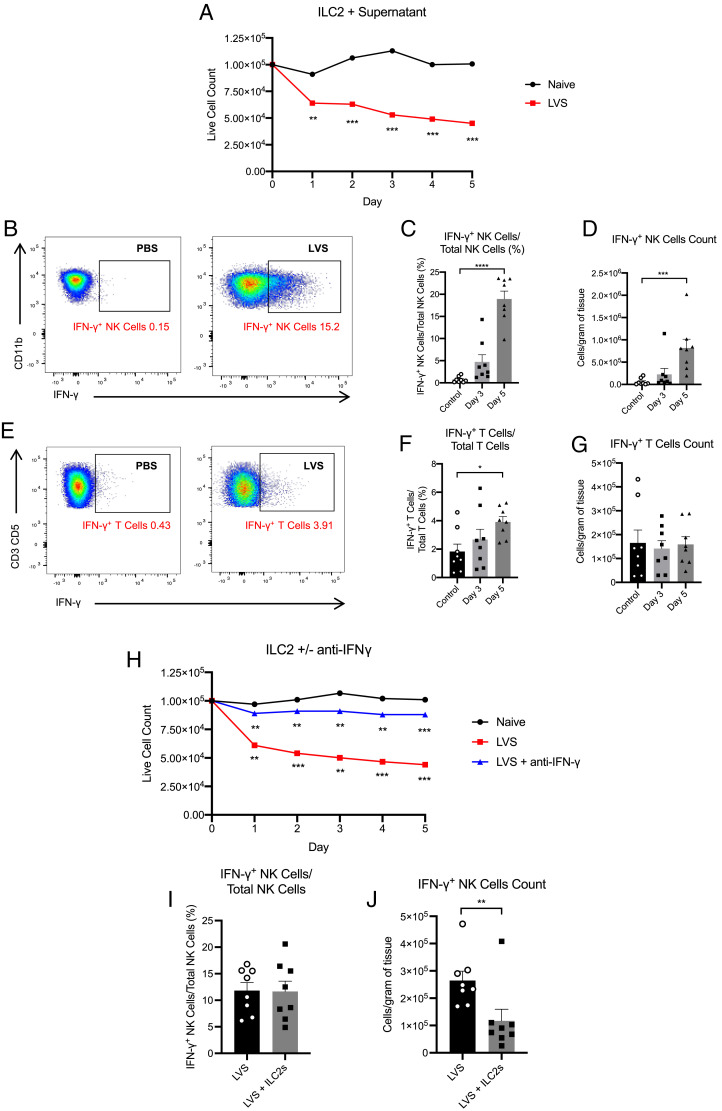
ILC2s are negatively regulated by IFN-γ from the LVS-infected lung. (**A**) FACS-sorted live CD45^+^Lin^−^CD90.2^mid/hi^CD127^+^KLRG1^+^ ILC2s (1 × 10^5^) were allowed to rest for 7 d in media with IL-7 (25 ng/ml) before culturing with either naive or *F. tularensis* LVS–infected cell-free lung supernatant. Data are representative of two independent experiments with cells pooled from four mice in each. (**B**) Representative plots for identification of NK cell (CD11b^+^NK1.1^+^T-bet^+^) IFN-γ production in PBS-treated (control) and *F. tularensis* LVS–infected WT mice at day 5 p.i. (**C** and **D**) IFN-γ production from cell populations was quantified and expressed as a frequency of total NK cells (C) and cell counts per gram of tissue (D). (**E**) Representative plots for identification of T cell (CD3^+^CD5^+^) IFN-γ production in PBS-treated (control) and *F. tularensis* LVS–infected WT mice at day 5 p.i. (**F** and **G**) IFN-γ production from T cells was quantified and expressed as a frequency of total T cells (F) and cell counts per gram of tissue (G). Data are from two independent experiments (*n* = 8). (**H**) Sort-purified ILC2s were cultured as in (A). ILC2s cultured with *F. tularensis* LVS–infected supernatant were cultured in the presence or absence of an anti–IFN-γ Ab. Data are representative of two independent experiments with cells pooled from four mice in each. (**I** and **J**) Frequency (I) and absolute cell numbers (J) of IFN-γ^+^ NK cells at day 7 p.i. in mice receiving PBS or purified ILC2s (as in (Fig. 3B). Data are pooled from two independent experiments (*n* = 8). Statistical analysis was performed using (A) paired *t* tests to compare data at each individual time point, (C and D) a Kruskal–Wallis test with Dunn’s correction for multiple comparisons, and (F and G) one-way ANOVA with Holm–Sidak’s correction for multiple comparisons. Statistical analysis in (H) was determined at each individual time point by repeated-measures one-way ANOVA with Holm–Sidak’s multiple comparison tests. Significance is shown as LVS-infected versus naive (below red line), and LVS-infected + anti-IFN-γ versus LVS-infected (below blue line); (I) Mann–Whitney *U* test; (J) unpaired *t* test. *p < 0.05, **p < 0.01, ***p < 0.001, ****p < 0.0001.

To this end, we developed an in vitro culture system where sort-purified ILC2s were cultured with either naive or LVS-infected cell-free lung supernatant. When ILC2s were cultured with lung supernatants from *F. tularensis* LVS–infected mice, the number of live ILC2s was significantly decreased over time compared with ILC2s cultured in lung supernatant from noninfected mice (Fig. 4A). Thus, these results suggest that infection with *F. tularensis* LVS induces the production of a soluble mediator that can reduce ILC2 numbers.

A hallmark of the immune response against *F. tularensis* LVS is the production of IFN-γ (20). As IFN-γ has previously been demonstrated to inhibit ILC2 function and activation (21, 22), and its production is enhanced after i.n. *F. tularensis* LVS infection (23), we next considered whether ILC2 numbers were reduced as a result of IFN-γ production during infection with *F. tularensis* LVS. Indeed, as previously described (23), we observed significantly enhanced production of IFN-γ by NK cells by day 5 p.i., both in frequency and cell number (Fig. 4B–D), a time point that coincides with the observed reduction in ILC2 numbers (Fig. 1E, 1F). Although the frequency of T cells producing IFN-γ was also elevated at day 5 p.i., there was no difference in the total numbers of T cells producing IFN-γ p.i. (Fig. 4E–G).

To determine whether this *F. tularensis* LVS–induced IFN-γ production was sufficient to drive a reduction in ILC2s, we performed our in vitro culture assay in the presence of an anti-IFN-γ–blocking Ab. Indeed, we found that blocking IFN-γ significantly reversed the effects of lung supernatant from *F. tularensis* LVS–infected animals in decreasing ILC2 numbers (Fig. 4H). Thus, taken together, these data suggest that the production of IFN-γ during infection with *F. tularensis* LVS contributes to the observed reduction in ILC2 numbers.

Our data suggest that expansion or transfer of ILC2s impedes effective immunity to *F. tularensis* LVS. Whereas IFN-γ may impair ILC2 responses, ILC2 expansion has been reported to suppress NK cell responses and protective type 1 immune responses (24). Thus, we next aimed to determine whether increasing ILC2 numbers prevented the production of early innate sources of protective IFN-γ. Thus, we analyzed IFN-γ^+^ NK cell levels after i.n. transfer of ILC2s and *F. tularensis* LVS infection. Although the proportion of cells producing IFN-γ was not altered by infection (Fig. 4I), there was a significant reduction in the total number of IFN-γ^+^ NK cells in the lung of infected mice that received transfer of ILC2s (Fig. 4J). Thus, taken together, these results suggest that bidirectional cross-regulation between IFN-γ–producing NK cells and ILC2s is a critical determinant of the immune control during the early stages of *F. tularensis* infection.

### Blockade of IL-5 reduces bacterial burden after *F. tularensis* LVS infection

**FIGURE 5.**
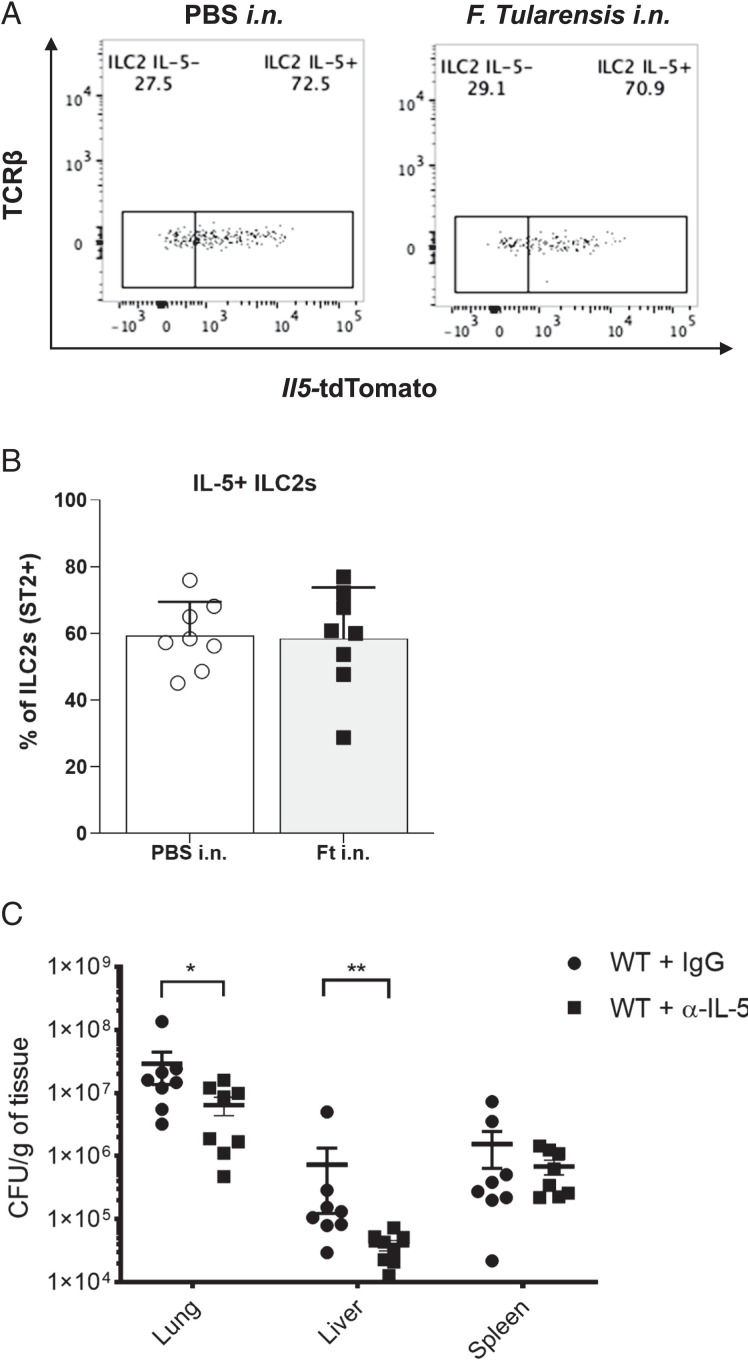
Ab-mediated blockade of IL-5 reduces bacterial burden in the lung and liver after *F. tularensis* LVS infection. (**A** and **B**) Red5 reporter mice, which a have targeted knock-in of the fluorescent reporter tdTomato downstream from the mouse *Il5* promoter (18), were infected with 1000 CFU of *F. tularensis* LVS i.n. or given PBS as a control. On day 5 p.i., cells were isolated from lungs, treated with PMA, ionomycin, and protein transport inhibitors, and expression levels of *Il5*-tdTomato in ILC2s were determined by flow cytometry. (A) Representative flow cytometry plots. (B) Data pooled from two independent experiments (*n* = 8). (**C**) C57BL/6 mice were injected i.p. with either 100 µg of control IgG or anti–IL-5 Ab 4 and 2 d before infection, infected with 1000 CFU of *F. tularensis* LVS i.n., and injected i.p. with control or anti–IL-5 Ab on the same day as infection, injected with Ab again at 2 d p.i., and then bacterial burdens in the lung, liver, and spleen of mice were analyzed at 4 d p.i. Data are pooled from two independent experiments (*n* = 8). Statistical analysis was performed using a Mann–Whitney *U* test (B and C). *p < 0.05, **p < 0.01.

Next, we sought to determine potential mechanisms by which ILC2s may suppress the control of bacterial burden p.i. with *F. tularensis* LVS. Following *F. tularensis* LVS infection, we detected only minimal expression of the type 2 cytokine IL-13 by ILCs (Supplemental Fig. 4A, 4B). In contrast, using Red5 reporter mice, which a have targeted knock-in of the fluorescent reporter tdTomato downstream from the mouse *Il5* promoter (18), we observed high frequencies of IL-5–producing ILC2s even in the lungs of both naive and infected mice (Fig. 5A, 5B). In contrast, we observed minimal expression of IL-5 by T cells in the lung in both naive mice and during infection (Supplemental Fig. 4C, 4D), indicating that the major source of IL-5 is ILC2 derived rather than adaptive cells at this time point. These data suggest that IL-5 production by ILC2s may be a potential mechanism by which these cells limit host clearance of bacteria during *F. tularensis* infection.

To determine whether early sources of IL-5 perturb host responses to *F. tularensis* LVS infection, we treated mice before and during infection with an anti–IL-5 Ab and measured bacterial burdens. We found that treatment with anti–IL-5 Ab caused a significant decrease in both pulmonary and liver bacterial burden compared with mice treated with control IgG (Fig. 5C), although no differences were observed in the spleen. Taken together with the data above, these results suggest that ILC2s may suppress bacterial clearance from the lung during *F. tularensis* infection at least in part via IL-5 production.

## Discussion

Much of the current understanding of the immune response during infection with *F. tularensis* has focused on the role of myeloid cells such as macrophages and neutrophils (6, 9), with the role of innate lymphocytes poorly defined. Despite the well-documented role for ILCs to respond rapidly to a variety of immunological challenges (10), infection with *F. tularensis* LVS only begins to impact the lung ILC compartment with a subset-specific reduction in ILC2s from day 5 p.i., associated with a concurrent increase in ILC1s. Indeed, these changes to the lung ILC compartment have been observed in the context of other intracellular infections, where plasticity and subsequent emergence of an ILC1-like IFN-γ–producing cell type via trans-differentiation of an ILC2 population was shown to be important in viral clearance (13). It is unclear whether the changes in the numbers of these specific ILC subsets represent a similarly plastic behavior by ILC2s during infection with *F. tularensis,* and whether the increase in ILC1s contributes to protective immunity. Evidence that would support the conversion of ILC2s to an ILC1 population in the context of *F. tularensis* infection is that IL-12 is readily detectable from 72 h p.i. with *F. tularensis* (25), and this cytokine is critical for the conversion of ILC2s to an ILC1-like phenotype (13). Conversely, infection with *F. tularensis* could instead propagate the expansion of a minor bona fide ILC1 population, given that IL-12 is also critical for the activation and expansion of ILC1s (10, 26). It is therefore important for future work to determine the exact relationship between ILC1s and ILC2s in the context of infection with *F. tularensis* LVS.

Of note, for consistency between experiments, we performed all infection experiments in female mice. Given that there have been reports of some sex differences in lung ILC2s (27–30), it will be interesting in future studies to determine whether the reduction of ILC2s during *F. tularensis* infection are sex specific.

ILC2s themselves are important in the immune response against several intracellular infections (11), with a loss of ILC2s significantly impacting both the integrity of airway epithelium and lung function during influenza infection (12). It is also interesting to note that p.i., ILC2s can provide a source of amphiregulin (AREG) for wound healing and tissue repair (12). However, given the lethality of infection with *F. tularensis* LVS in mice (15), the role of ILCs in the resolution phase cannot be examined in this particular model. ILC2s can also play a detrimental role in the control of pulmonary infections, contributing to the exacerbation of tissue pathology (11). Indeed, activation of ILC2s results in a strong type 2 immune response characterized by production of the effector cytokines IL-5 and IL-13 (10). Specifically, ILC2-derived IL-13 drives airway hyperreactivity during multiple viral infections (31–33). Interestingly, IL-13 can induce an alternative activation state in macrophages in the context of infection with *F. tularensis* LVS, resulting in enhanced intracellular replication and survival of bacteria (34). However, our data show that ILCs produce minimal IL-13 during *F. tularensis* LVS infection, suggesting that other alternative cytokines are responsible for the immunoregulatory role of ILC2s in this context, and that non-ILC2 sources of IL-13 are important in promoting alternative activation of macrophages.

In contrast, we show that ILC2s produce IL-5 both at homeostasis and during *F. tularensis* LVS infection, and that neutralization of IL-5 leads to enhanced bacterial burdens following infection. These data suggest that IL-5 may drive suppression of protective immunity. It has previously been shown that ILC2-derived IL-5 supports the homeostatic function of eosinophils (18), and activated ILC2s can drive the induction of eosinophilic airway inflammation (35). Interestingly, ILC2-derived IL-5 and activation of eosinophils can inhibit the production of IFN-γ and the cytotoxic function of NK cells in antitumor immune responses (24). Thus, it is possible that ILC2-derived IL-5 acts to limit type 1 immunity during *F. tularensis* LVS infection via effects on eosinophils that then suppress NK cells, although more work is required to investigate this possibility. A potential role for ILC2s and IL-5 during *F. tularensis* LVS infection is in line with a recent study suggesting their importance in regulation of IgM production by B cells in responses to *F. tularensis* LVS–derived LPS vaccination in murine models (36).

A hallmark of infection with *F. tularensis* is the production of IFN-γ (20), which is known to inhibit ILC2 function and proliferation (21, 37, 38). More specifically, mice with elevated levels of constitutive IFN-γ display diminished ILC2 responses during infection with the nematode *Nippostrongylus brasiliensis* (21). This IFN-γ–mediated effect is also observed during coinfection with *N. brasiliensis* and *Listeria monocytogenes*, a bacterium that provokes a strong IFN-γ–mediated response (20). Furthermore, the administration of IFN-γ during rhinovirus infection significantly attenuates immunopathology, diminishing the output of IL-13 from ILC2s (39). We now reveal that IFN-γ also drives an *F. tularensis* infection–induced reduction in ILC2s. Enhanced levels of IFN-γ observed at day 5 p.i. in our model coincide with the reduction in ILC2 numbers in vivo, which, coupled with our in vitro data showing that IFN-γ can reduce ILC2 numbers, highlights the importance of this cytokine in control of ILC2s during *F. tularensis* LVS infection. Nevertheless, other cytokines such as the type 1 IFNs and IL-27 can also inhibit ILC2 function and survival (20, 30). Thus, future work is required to determine whether other soluble mediators can also impact ILC2 numbers during *F. tularensis* infection.

Taken together, our findings suggest that ILC2s in the lung may impair optimal immunity to *F. tularensis* LVS. Numbers of ILC2s are reduced during infection, which may be a result of cross-regulation with the protective type 1 immune response that acts to reduce the potentially detrimental role of ILC2s. Mechanistically, our data suggest a potential cross-regulation between NK cells and IFN-γ and ILC2s and IL-5 in determining the magnitude of *F. tularensis* LVS bacterial burdens during the early stages of infection, with perturbation of this axis via cytokine neutralization or manipulation of ILC2 abundance having dramatically altered the pathogen burden.

Overall, our data begin to highlight the potentially detrimental role of ILC2s in the control of infection with *F. tularensis*. Future work focusing on how this subset-specific reduction in ILC2s occurs may allow for a more targeted approach of manipulation of ILC2 numbers. Although more work is required to determine the overall outcome of depleting ILC2s on the broader immune response and the host, our work highlights that early depletion of ILC2s is an important pathway to explore further with the aim of promoting a more beneficial host immune response against *F. tularensis*.

## Supplementary Material

Supplemental 1 (PDF)
