# Supplementary material for: Group 2 Innate Lymphoid Cells Are Detrimental to the Control of Infection with Francisella tularensis
Source: J Immunol. 2023 Jan 16;210(5):618–27. doi: 10.4049/jimmunol.2100651 (PMC9946898; doi:10.4049/jimmunol.2100651)
Supplement: Supplemental 1 (PDF) [file JI_2100651_Supplemental_1.pdf]

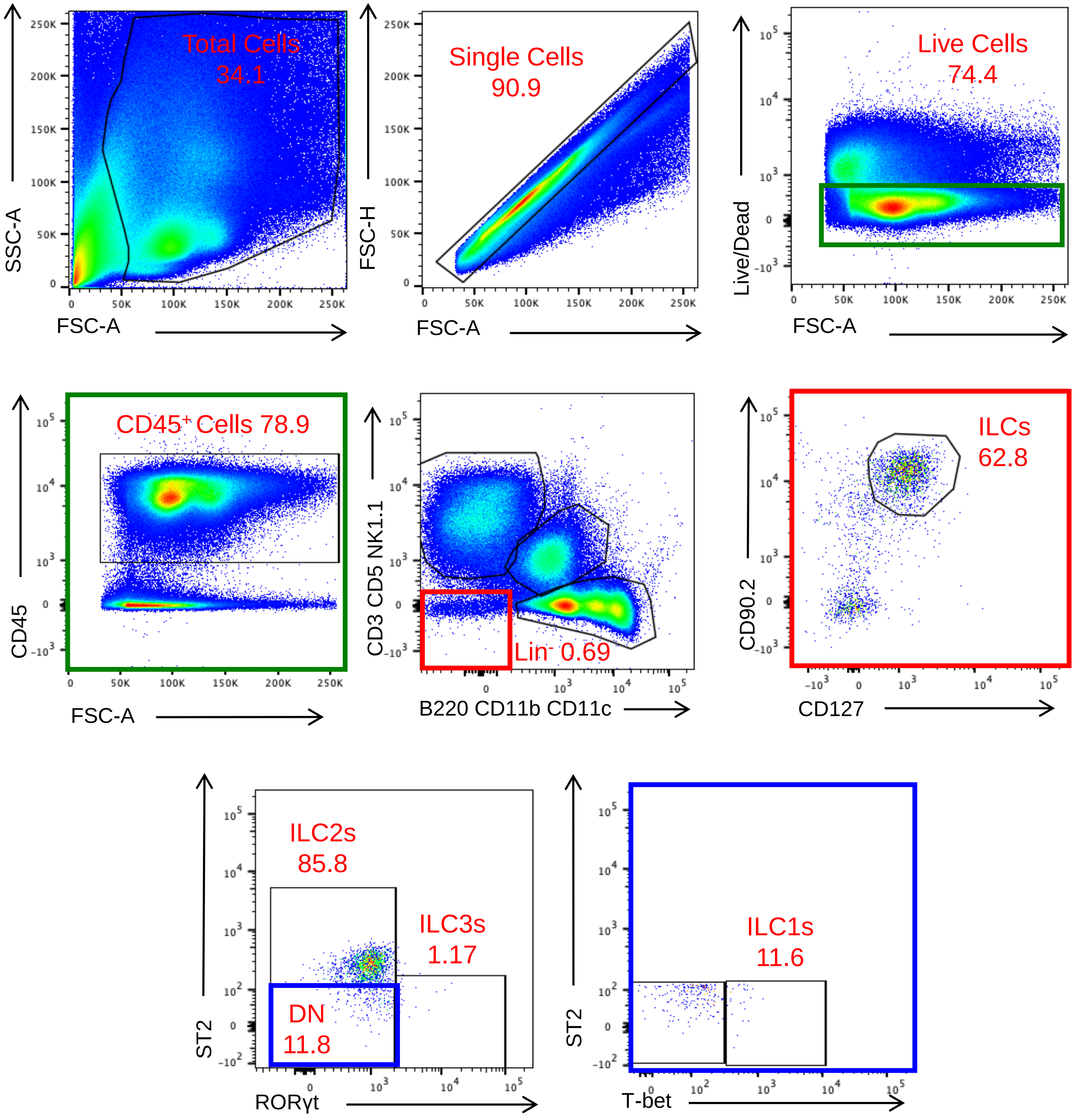

Supplementary figure 1: Gating strategy for identification of ILC subsets from the lung. Lung cells were isolated as described in materials and methods. Representative plots are from an uninfected C57BL/6 mouse, with cells gated first on forward scatter area (FSC-A) and side scatter area (SSC-A), then on single cells via FSC-A and FSC-height (FSC-H). Dead cells were excluded via staining with a Live/Dead dye, $CD45^{+}$ cells gated and then Lineage-negative (CD3, CD5, NK1.1, B220, CD11b, CD11c-negative) cells carried forward. ILCs were gated as $CD90.2^{+}$ $CD127^{+}$ cells, then split into ILC2s via ST2 staining and ILC3s via RORγt staining. ILCs staining for neither marker (double negative, DN) were further gated for T-bet for identification of ILC1s.

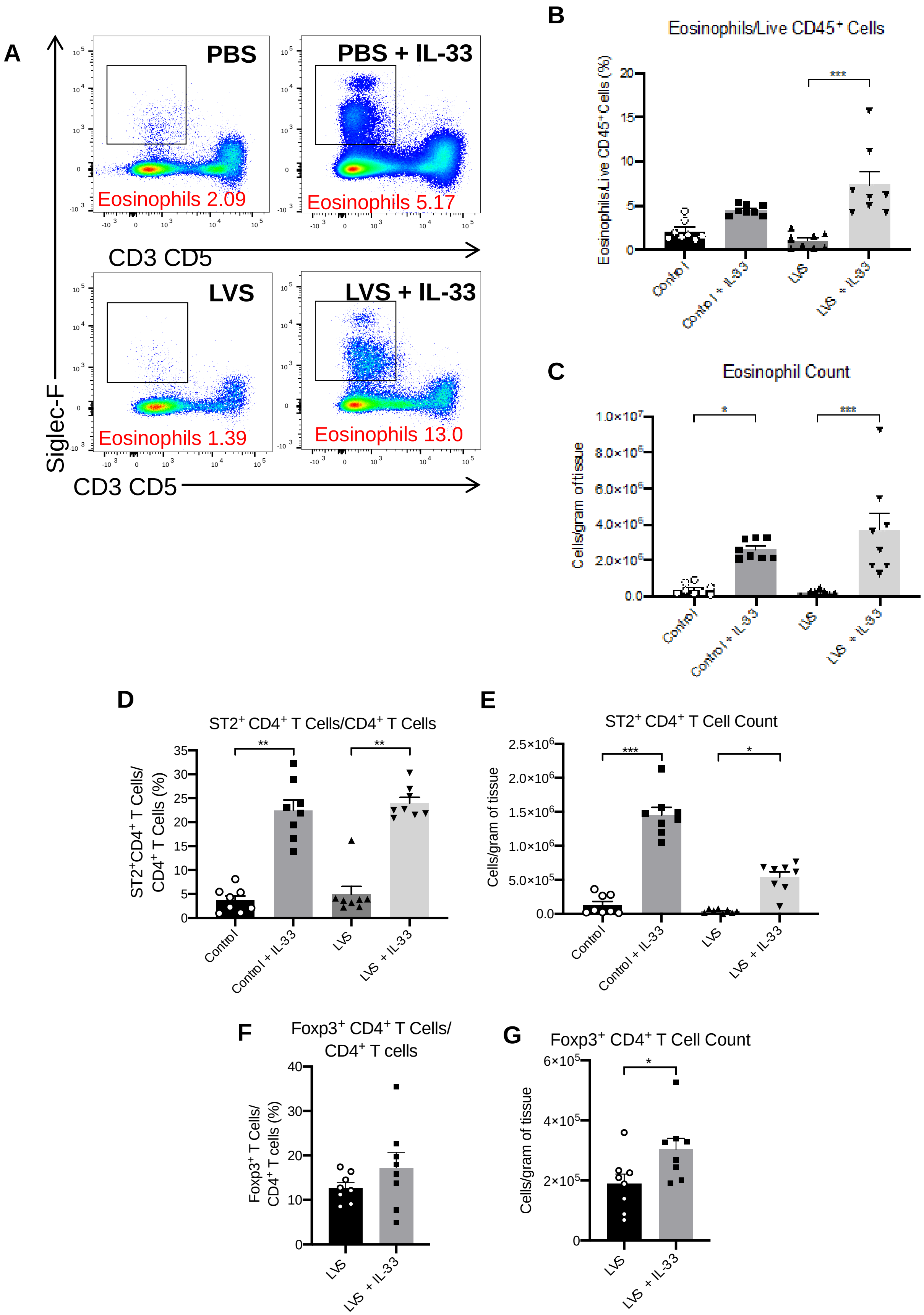

Supplementary figure 2: IL-33 treatment impacts on multiple immune cell populations during infection with *F. tularensis* LVS. (A) Representative plots for identification of eosinophils (live $CD45^+Ly6G^-CD64^-Siglec\text{-}F^+$) in untreated or IL-33-treated mice during infection with *F. tularensis* LVS. **(B-C)** Eosinophils, **(D-E)** $ST2^+$ $CD4^+$ T cells (live $CD45^+CD3^+CD5^+$) and **(F-G)** Foxp3+ regulatory T cells (live $CD45^+CD3^+CD5^+CD4^+Foxp3^+$) were quantified and expressed as a frequency of live $CD45^+$ cells for $ST2^+$ $CD4^+$ T cells (**D**) or total $CD4^+$ T cells for regulatory T cells (**F**), and cell counts per gram of tissue (**E+G**). Data represents two independent experiments (n=8). Statistical analysis was performed using **(B-E)** Kruskal Wallis test and **(F-G)** unpaired t-test. Dunn's multiple comparisons tests were performed for data in **(B-E)**. * $p<0.05$; ** $p<0.01$ *** $p<0.001$.

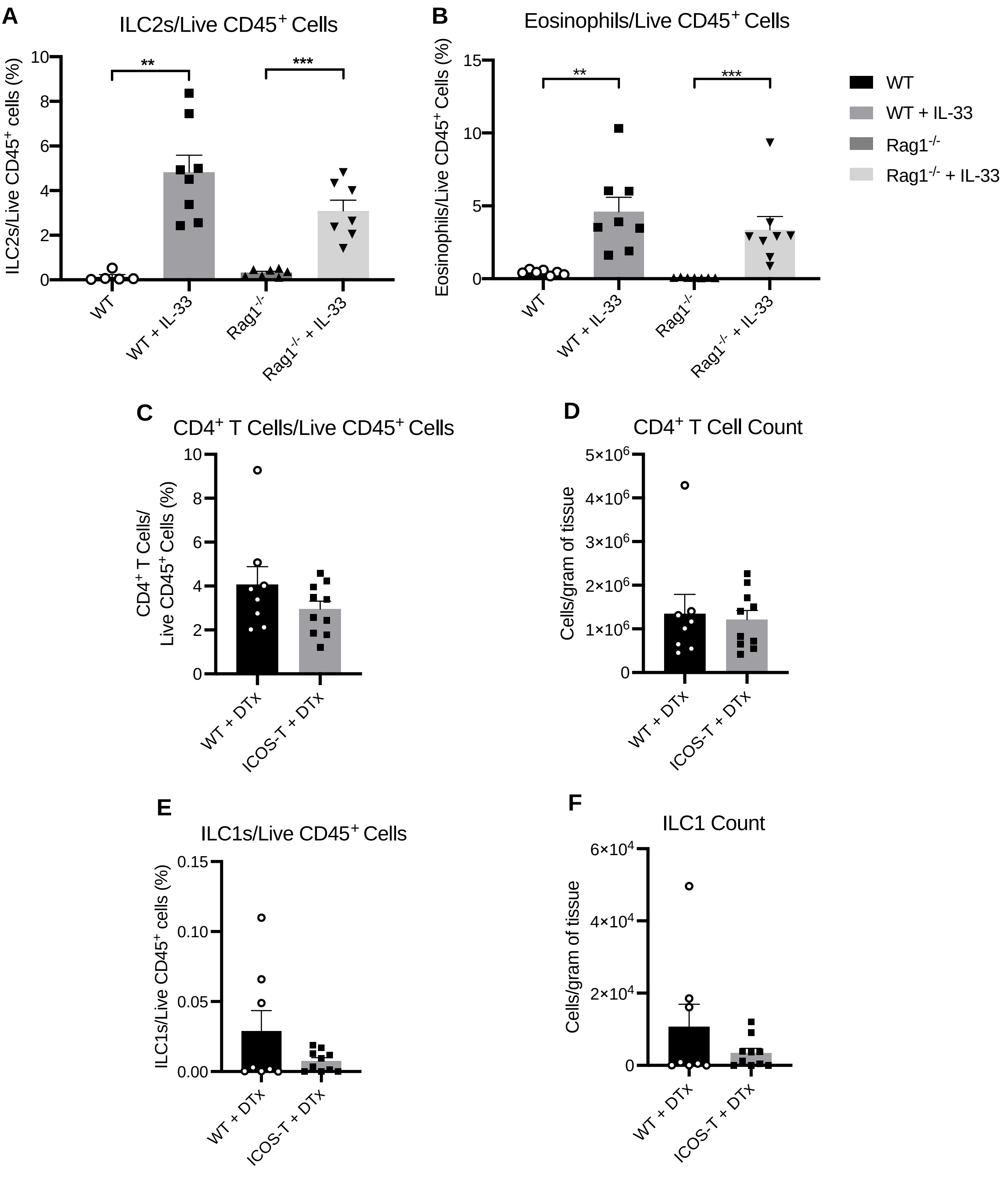

Supplementary figure 3: IL-33-treated Rag1$^{-/-}$ mice display increased percentages of ILC2s and eosinophils during infection with *F. tularensis* LVS, and ILC1 and CD4^{+} T cell frequency and numbers are unaltered in ILC2-depleted mice. (**A**) ILC2s and **(B)** eosinophils (live CD45^{+}Ly6G^{-}CD64^{-}Siglec-F^{+}) were identified in untreated or IL-33-treated mice during infection with 100 CFU *F. tularensis* LVS. Cell populations were quantified and expressed as a frequency of live CD45^{+} cells. Data represents two independent experiments (n=5-8). (**C-F**) iCOS-T mice (17) or littermate controls without Cre or DTR expression (WT) were injected with DT and infected with 1000 CFU *F. tularensis* LVS, as shown in figure 3C. Lung cells were isolated and analysed for flow cytometry for CD4^{+} T cells and ILC1s. (**C**) Frequency as a % of CD45+ cells and (**D**) absolute cell number per g of tissue for CD4^{+} T cells; (**E**) Frequency as a % of CD45+ cells and (**F**) absolute cell number per g of tissue for ILC1s. Data pooled from 2 independent experiments (n = 8-10). Statistical analysis in **A** and **B** was performed between untreated and treated groups for each genotype respectively, using an unpaired t-test for parametric data, or Mann-Whitney for non-parametric data; **p<0.01;***p<0.001.

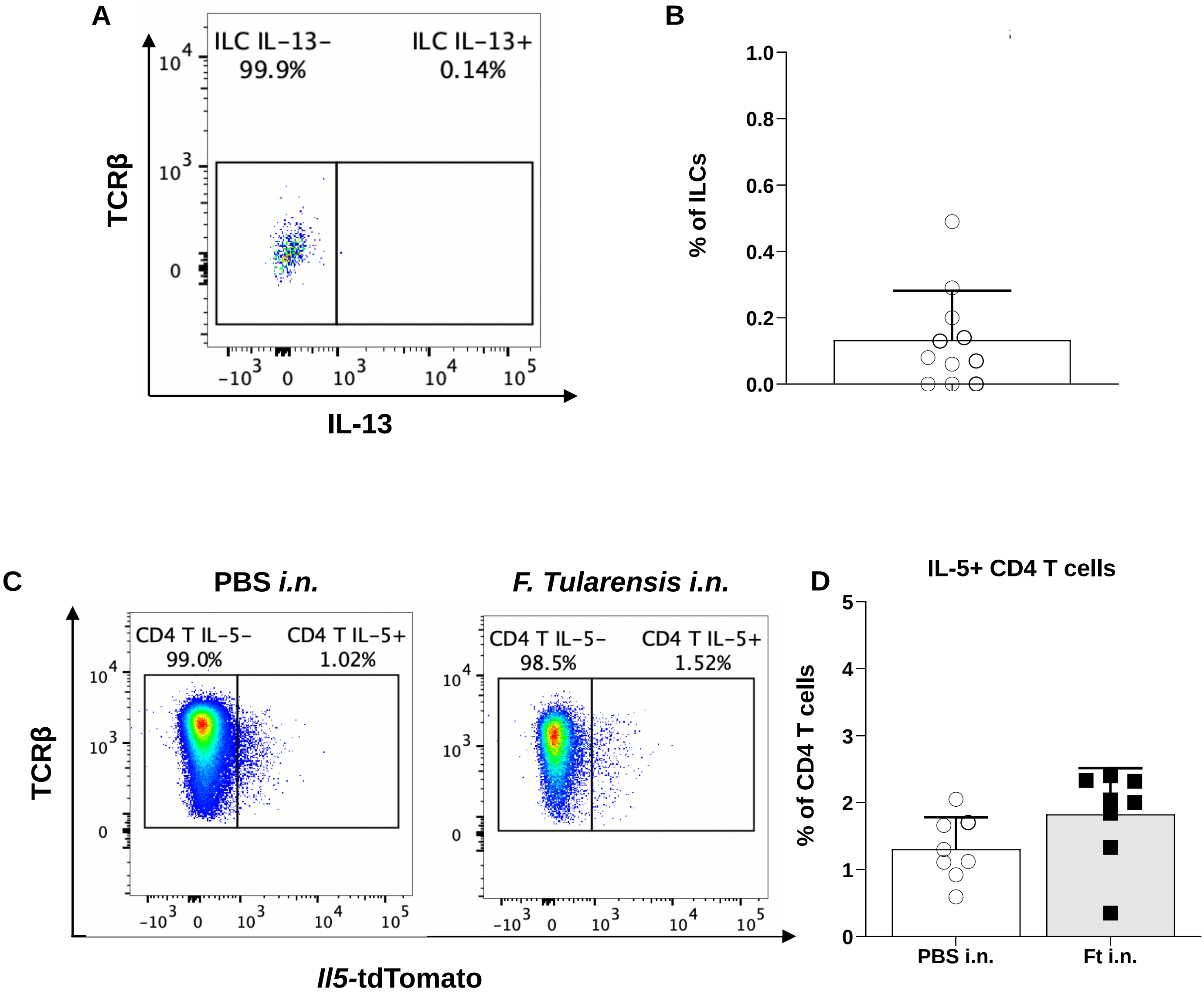

Supplementary figure 4: Minimal expression of IL-13 by ILCs and IL-5 by CD4$^+$ T cells after infection with *F. tularensis* LVS. (**A-B**) C57BL/6 mice were infected with 1000 CFU *F. tularensis* LVS *i.n.*, lung cells isolated at day 5 p.i, treated with PMA, ionomycin and protein transport inhibitors and expression of IL-13 in total ILCs determined by flow cytometry. (**A**) shows representative flow cytometry plots, (**B**) shows data pooled from 2 independent experiments (n = 11). (**C-D**) Red5 reporter mice were infected with 1000 CFU *F. tularensis* LVS *i.n.*, lung cells isolated at day 5 p.i, treated with PMA, ionomycin and protein transport inhibitors and expression of *Il5*-tdTomato in CD4$^+$ T cells determined by flow cytometry. (**C**) shows representative flow cytometry plots, (**D**) shows data pooled from 2 independent experiments (n = 8).
